# The multifaceted roles of FOXM1 in pulmonary disease

**DOI:** 10.1186/s12964-019-0347-1

**Published:** 2019-04-16

**Authors:** Yumei Li, Feng Wu, Qi Tan, Mengfei Guo, Pei Ma, Xuan Wang, Shuai Zhang, Juanjuan Xu, Ping Luo, Yang Jin

**Affiliations:** 1 Department of Respiratory and Critical Care Medicine, NHC Key Laboratory of Pulmonary Diseases, Union Hospital, Tongji Medical College, Huazhong University of Science and Technology, 1277 Jiefang Avenue, Wuhan, 430022 China; 2 Center for Translational Medicine, Union Hospital, Tongji Medical College, Huazhong University of Science and Technology, 1277 Jiefang Avenue, Wuhan, 430022 China

**Keywords:** FOXM1, Transcriptional regulation, Cell cycle, Cell proliferation, Target therapy, Pulmonary disease

## Abstract

Forkhead box M1 (FOXM1), a transcriptional regulator of G1/S and G2/M transition and M phase progression in the cell cycle, plays a principal role in many physiological and pathological processes. A growing number of studies have focused on the relationship between abnormal FOXM1 expression and pulmonary diseases, such as lung cancer, chronic obstructive pulmonary disease (COPD), asthma, acute lung injury (ALI), pulmonary fibrosis, and pulmonary arterial hypertension (PAH). These studies indicate that the FOXM1 regulatory network is a major predictor of poor outcomes, especially in lung cancer, and provide novel insight into various pulmonary diseases. For the first time, this review summarizes the mechanistic relationship between FOXM1 dysregulation and pulmonary diseases, the benefits of targeting abnormal FOXM1 expression, and the questions that remain to be addressed in the future.

**Electronic supplementary material:**

The online version of this article (10.1186/s12964-019-0347-1) contains supplementary material, which is available to authorized users.

## Background

FOXM1 is a member of the FOX family of transcription factors, which share a preserved winged-helix DNA-binding domain. FOXM1 was previously known in the literature as Trident (in mouse), HFH-11 (in human), WIN or INS-1 (in rat), MPP-2 (partial human cDNA) or FKHL-16 [1]. The FOXM1 gene maps to human chromosome 12p13.3 and encodes 747 amino acids [2]. Due to the alternative splicing of exons, four common distinct isoforms are generated: FOXM1a, FOXM1b, FOXM1c and FOXM1d. All Foxm1 subtypes are active transcription factors except FOXM1a, which lacks transactivation activity [3, 4]. To date, the majority of studies have focused on the active type FOXM1b; in this review, we refer to different active isoforms as simply FOXM1.

FOXM1 functions as a transcriptional regulator of the cell cycle and chromatin assembly and exhibits a spatiotemporal expression pattern during growth and development depending on the organ, tissue, and cell specificity. FOXM1 interacts with multiple signaling pathways and directly or indirectly activates the transcriptional expression of target genes, which are involved in many aspects of cell fate in physiological and pathological processes [4]. The following evidence supports the above viewpoints: (i) For the first time, FOXM1, from the murine thymus, was identified as a novel member of the forkhead/winged-helix family that functions as a transcriptional factor involved in cell cycle-specific gene regulation [5]. (ii) Subsequently, Ye H and colleagues indicated that FOXM1 is broadly expressed in mouse embryonic cells, such as in the mesenchymal and epithelial cells of the liver, lung, intestine, renal cortex, and urinary tract, but its expression is restricted to a few types of proliferative cells in adult tissues; in addition, its expression is reactivated in adult cells via proliferative signals released during injury and repair or tumorigenesis [6]. (iii) Kalinichenko VV first reported that FOXM1 expression is induced in response to lung injury throughout the lung repair process [7]. (iv) FOXM1 is frequently upregulated in 22 human malignancies, including lung cancer, and the FOXM1 regulatory network is a major predictor of poor outcomes [8]. After 20 years of research, FOXM1 was shown to be closely related to pulmonary diseases.

As a result of harmful environmental exposure and genetic differences, a group of pulmonary diseases, including lung cancer, chronic obstructive pulmonary disease (COPD), asthma, acute lung injury (ALI), pulmonary fibrosis, and pulmonary arterial hypertension (PAH), contribute to progressive damage characterized by the destruction of lung structure, the disruption of gas exchange and even death from respiratory failure. These pulmonary diseases cause significant morbidity and mortality worldwide [9]. Several studies have shown that FOXM1 is involved in pulmonary diseases. This review will focus on the known and unknown information regarding the transcriptional relationship between FOXM1 dysregulation and lung diseases, aiming to provide an overview of insights into the management of pulmonary diseases and attempting to highlight the underlying research questions regarding the role of FOXM1 in pulmonary diseases that should be addressed in the future.

## Forkhead box M1 (FOXM1): a brief overview

The FOXM1 transcription factor is recognized as a well-defined regulator of cell cycle progression, as it is critical for G1/S and G2/M transition and M phase progression. Several key mitotic regulatory genes, such as Skp2 and Cks1, which degrade p21^Cip1^ and p27^Kip1^, as well as cyclin A, cyclin B, polo-like kinase1 (PLK1), Cdc25A, Cdc25B, Cdc25C, Aurora B kinase (AURKB), survivin, centromere protein A (CENPA) and CENPB, are under the transcriptional control of FOXM1 [10, 11]. On the other hand, FOXM1 also positively autoregulates its own transcription [12].

In addition to cell cycle progression, FOXM1 regulates many aspects of biological progression, including cell proliferation, cell renewal, cell differentiation, DNA damage repair, tissue homeostasis, cell migration, angiogenesis and cell survival [11]. Evidence that FOXM1 is critical for proper lung development is shown below: (i) FOXM1 is required for perinatal lung function and directly transcriptionally activates the Sftpb and Sftpa promoters [13]. (ii) FOXM1 mediates cross talk between the Kras/mitogen-activated protein kinase and canonical Wnt/β-catenin pathways of basal cells and embryonic respiratory epithelia during proper lung development [14, 15]. (iii) FOXM1 is critical for the differentiation and maintenance of epithelial cells lining conducting airways [16]. (iv) FOXM1 is essential for embryonic pulmonary vasculature development [17, 18]. FOXM1 is critical for cell proliferation and required for proper embryonic lung development, suggesting that this proliferation pathway present during normal development could be reawakened in adult diseases characterized by aberrant proliferation.

## The role of FOXM1 in lung cancer

Lung cancer is the most frequently diagnosed cancer, accounting for approximately 11.6% of all cancers diagnosed worldwide, and the leading cause of cancer death (18.4% of total cancer deaths) [19]. Several studies have demonstrated that numerous oncogenic stimuli that initiate different signaling cascades ultimately converge on a common program targeting FOXM1 transcription factor activity, resulting in the upregulation of FOXM1 in lung cancer [20]. FOXM1 is a promising candidate for use as a diagnostic biomarker [21, 22] and treatment target in lung cancer [23, 24]. Additionally, abnormal upregulation of FOXM1 is associated with poor clinical outcomes for patients with lung cancer. Mechanically, the dysregulation of FOXM1 consequently culminates in abnormalities in the cancer cell proliferation, replication, invasion, metastasis and apoptosis programs, which together contribute to the development of lung cancer and mediate drug resistance [20] (Fig. 2).

### Expression and regulatory network of FOXM1 in lung cancer

**Fig. 1:**
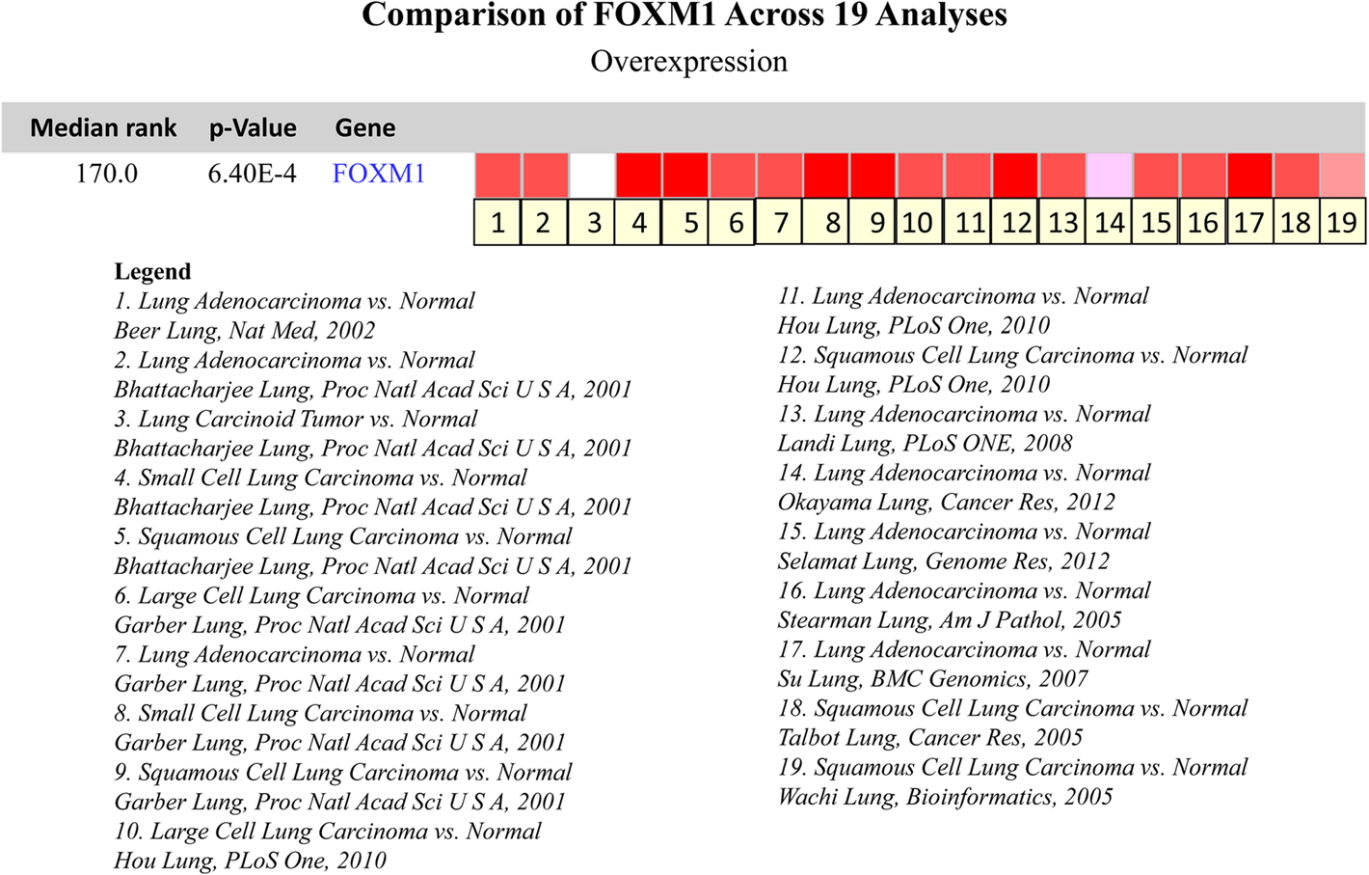
Expression of FOXM1 of lung cancer in studies identified in the Oncomine database. One through nineteen represent the 19 studies on the expression of FOXM1 in lung cancer. The darker red indicates higher FOXM1 expression in the chips

FOXM1 cDNA expression is increased > 2-fold in squamous cell carcinoma and adenocarcinoma of the lung [25]. FOXM1 mRNA data related to lung cancer were retrieved from the Oncomine database [26, 27], and 19 studies involved the FOXM1 mRNA profiles in lung cancer tissues and normal tissues, including 1197 clinical samples in total. Overall, FOXM1 mRNA expression in lung cancer is higher than that in normal tissues (Fig. 1). FOXM1 protein levels are increased in adenocarcinoma, squamous cell carcinoma, adenosquamous carcinoma, large cell neuroendocrine carcinoma (LCNEC) and small cell neuroendocrine carcinoma (SCLC) compared with those in corresponding normal lung tissues [21, 25, 28, 29]. In summary, FOXM1 expression is increased in lung cancer tissues with different histological subtypes at the mRNA and protein levels.

**Fig. 2:**
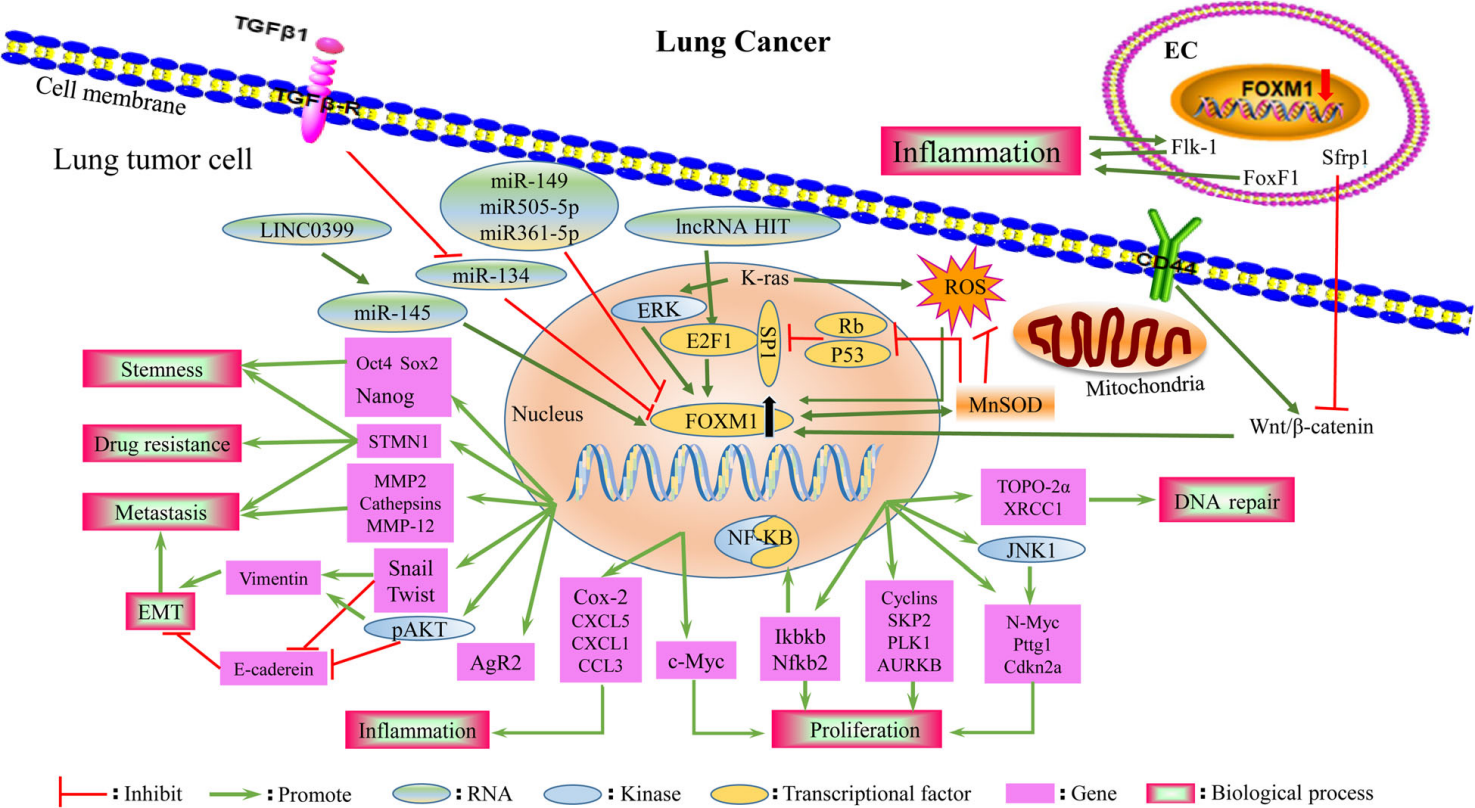
The FOXM1 regulatory network of lung cancer. Numerous oncogenic stimuli that initiate different signaling cascades ultimately contribute to a common program targeting FOXM1 transcription factor activity in lung tumor cells. FOXM1 transcriptional factor triggers various genes involved in many biological process, consequently facilitating tumor cells proliferation, DNA repair, invasion and metastasis, stemness and drug resistance, which altogether contribute to the development of lung cancer

The expression of FOXM1 is increased by oncoproteins (such as Kras, EGFR, MYC, YAP, and AKT), whereas FOXM1 expression is decreased by tumor suppressors (such as miRNAs, RB, P53, and FOXO3a) [30]. The induction of FOXM1 is driven by oncoproteins (Kras, E2F1, AKT) and repressed by tumor suppressors (miRNAs, RB, P53) in lung cancer, and the roles of FOXM1 in oncogenic pathways vary. The FOXM1 regulatory network in lung cancer is summarized in Fig. 2. FOXM1 functions downstream of oncogenic Kras and is required for its induction of lung cancer formation [24]. FOXM1 acts as a target gene of E2F1, mediating the non-small cell lung cancer (NSCLC) proliferation initiated by the lncRNA-HIT–E2F1-FOXM1 axis [31]. Tyrosine kinase inhibitors (TKIs) contribute to the aberrant activation of the AKT/FOXM1 pathway during the lung cancer treatment process [32]. Increased FOXM1 expression occurs secondary to reduced miR-145 expression, facilitating NSCLC tumorigenesis via the LINC00339/miR-145/FOXM1 axis [33]. miR-509-5p, miR-149 and miR-361-5p act as tumor suppressors, directly binding to the FOXM1 promoter and downregulating its expression in human NSCLC [34–36]. FOXM1 is also upregulated by manganese superoxide dismutase (MnSOD) through reducing p53 and RB expression, which consequently derepresses Sp1 expression and releases E2F1 from the RB–E2F1 complex, promoting the binding of E2F1 to FOXM1 promoter-binding sites [37]. The expression of FOXM1 is increased in response to high levels of reactive oxygen species (ROS) induced by sulfur mustard (SM) and may be associated with an increased risk of lung cancer among SM-exposed patients [38]. These data provide evidence that ROS are required for the induction of FOXM1 by oncogenic Kras and that elevated FOXM1, in turn, downregulates ROS levels by directly stimulating the expression of ROS scavenger genes (such as MnSOD), allowing tumor cells to escape premature senescence and apoptosis [39]. A previous study demonstrated the transcription factor FOXM1 to be the downstream target of the Sonic Hedgehog (SHH) effector Gli1 transcription factor in basal cell carcinomas [40]. Hedgehog signaling was activated in NSCLCs, and several Hedgehog components, including PTCH1, SMO, and GLI1, were correlated with the increased expression of FOXM1, indicating that overexpression of FOXM1 may be a new mechanism by which SHH signaling induces cell proliferation in NSCLC; however, these results need to be investigated further [41].

### FOXM1 correlates with lung cancer diagnosis, treatment and poor outcomes

With most problems stemming from discriminating the subtypes of pulmonary neuroendocrine tumors, the combinations of FOXM1 with its downstream targets p27kip1 and p21waf1/cip1 may serve as an ancillary test to enhance diagnostic accuracy. Distinguishing LCNECs from SCLCs with the combination of FOXM1(+)/p27kip1(high) and discriminating atypical carcinoids from typical carcinoids with the combination of FOXM1(−)/p21waf1/cip1(−) have the best sensitivity [21]. FOXM1 is expressed more frequently in high-grade pulmonary neuroendocrine tumors than in carcinoid tumors [21]. This research is consistent with the results shown in Fig. 1, which show that FOXM1 is expressed at higher levels in SCLC than in carcinoid tumors. Moreover, FOXM1 directly binds to and transcriptionally activates the human AGR2 gene promoter, mediating the induction and maintenance of pulmonary invasive mucinous adenocarcinoma from benign adenomas in vivo [42].

Interestingly, Tang et al. identified FOXM1 as a diagnostic marker for discriminating benign and malignant pleural effusion. Moreover, FOXM1 expression in the metastatic pleural effusion from the group with a cytology diagnosis was higher than that from the group without a cytology diagnosis [22].

A previous study showed that patients with stage I lung cancer who smoked or were greater than 55 years of age, strong FOXM1 expression had a substantially higher recurrence rate than those with weak/negative FOXM1 expression. Thus, adjuvant therapy including chemotherapy might be beneficial for these patients. If strong FOXM1 expression is confirmed in surgical patients that have early stage I/II cancer and no lymph node metastasis or well/moderately differentiated carcinoma, adjuvant therapy and frequent follow-up might be suggested [43]. FOXM1 expression can predict the sensitivity to cisplatin-based chemotherapy in advanced stage IIIB/IV NSCLC patients. The cisplatin-based chemotherapy response rate of patients with FOXM1-positive tumors is lower than that of patients with FOXM1-negative tumors [23].

FOXM1 overexpression was significantly associated with a high TNM stage, lymph node metastasis, patients with a history of smoking [29], poor tissue differentiation, poor disease-free survival [28, 43], low response rate, poor progression-free survival, overall survival [23], and poor relapse-free survival [37]. FOXM1 is an independent and significant prognostic indicator and recurrence index for patients with NSCLC [23, 28, 29, 32, 37, 43].

### FOXM1 promotes pulmonary tumorigenesis by sustaining proliferation

FOXM1 is abundantly expressed in highly proliferative human NSCLC [25, 41] as well as in urethane- or 3-methylcholanthrene (MCA)/butylated hydroxytoluene (BHT)-induced mouse lung tumors [44]. The FOXM1 transcription factor stimulates the proliferation of tumor cells during the progression of NSCLC [25]. In addition, treatment of Rosa26-FOXM1 transgenic mice, which overexpress FOXM1 in all tissues, with MCA/BHT, a lung tumor initiation/promotion protocol, induced the formation and increased the number and size of lung tumors as well as significantly increased the expression of cell cycle genes, DNA replication and the expression of cyclooxygenase-2 (Cox-2), which is associated with focal persistent inflammation, leading to increased macrophage infiltration and increased levels of the chemokine ligands CXCL5, CXCL1 and CCL3, cathepsins and matrix metalloprotease-12 (MMP-12). FOXM1 directly binds to the human Cox-2 promoter and induces its expression in tumor and nontumor cells in inflammatory regions of injured lungs [44]. FOXM1 is required for the Kras-induced formation of lung cancer by activating genes critical for the nuclear factor-kB (NF-kB) and c-Jun N-terminal kinase (JNK) pathways, such as Ikbkb, Nfkb1, Nfkb2, Rela, Jnk1, N-Myc, Pttg1 and Cdkn2a. In addition, FOXM1 directly binds to promoter regions of Ikbkb, Nfkb2, N-Myc, Pttg1 and Cdkn2a [24]. This report is consistent with a previous study showing that FOXM1-ΔN accelerated tumor growth induced by activated Kras in epFOXM1/ep Kras mice [16].

Deletion of FOXM1 in vitro reduced the expression of cell cycle-promoting cyclin A2 and cyclin B1 genes, diminished DNA replication (by directly binding to the promoter region of TOPO-2a) and attenuated mitosis. Urethane- or MCA/BHT-treated Mx-Cre FOXM1−/− mice and transgenic (epFOXM1−/−) mice exhibited diminished proliferation of lung tumor cells, causing a striking reduction in the number and size of lung tumors. Moreover, conditional deletion of FOXM1 dramatically decreased lung tumor progression in preexisting lung tumors [25, 45]. Moreover, epKrasG12D/epFoxm1−/− mice (deletion of FOXM1 but activation of KrasG12D in epithelial cells) exhibited dramatically reduced numbers and sizes of lung tumors [24].

Taken together, these results suggest that the abnormal transactivation of FOXM1 is associated with lung cancer initiation and progression. However, another recent study also demonstrated that endothelial-specific expression of FOXM1 inhibits lung cancer growth by limiting lung inflammation and inhibiting canonical Wnt/β-catenin signaling in lung alveolar type II epithelial cells. FOXM1 directly activates the Flk-1 and FoxF1 promotors to limit lung inflammation, and the secreted frizzled-related protein 1 (Sfrp1) promotor inhibits canonical Wnt/β-catenin signaling in lung alveolar type II epithelial cells. Furthermore, genetic deletion of FOXM1 from endothelial cells (Tie2-Cre/FOXM1^fl/fl^ or enFOXM1−/− mice) increased the numbers and diameters of lung tumors [46]. Interestingly, this finding is consistent with that of previous work, showing that increased FOXM1 expression inhibits canonical Wnt/β-catenin signaling through direct transcriptional activation of Jnk1 and Axin2 in embryonic respiratory epithelial cells [14]. These data suggest that in certain cell contexts, FOXM1 may act as a tumor suppressor by indirectly suppressing the Wnt pathway. This phenomenon can be explained by the fact that the functions of FOXM1 change based on cell type (Table 1).

**Table 1:** The targets and functions of FOXM1 during the development of pulmonary disease

Cell types	Direct targets	Regulatory role	Biological process	References
Alveolar epithelial cells	Cox-2	Activate	Prostaglandin synthesis Pulmonary inflammation	[44]
	Stfpa Stfpb	Activate	Surfactant production Differentiation	[13]
	Jnk1 Axin2	Activate	Canonical Wnt signaling	[14]
Airway epithelium cells	Spdef	Activate	Mucus production Differentiation	[65]
Clara cells	Sox-2 Scgb1a1	Activate	Differentiation	[67]
Cancer cells (epithelial origin)	TOPO-2a	Activate	DNA repair	[45]
	STMN1	Activate	Metastasis	[32]
	Snail1	Activate	EMT	[9]
	MMP2	Activate	Metastasis	[37]
	Vimentin MMP-9	Activate	EMT	[29, 58]
	E-cadherin	Inhibit	EMT	[29, 58]
	Twist	Activate	EMT	[47]
	Ikbkb Nfkb2	Activate	NF-KB pathway	[24]
	N-Myc Pttg1 Cdkn2a	Activate	JNK pathway	[24]
Endothelial cells	Ctnnb1(β-catenin)	Activate	Adherens junctions	[80]
	Flk-1 FoxF1	Activate	Vascular formation Pulmonary inflammation	[46]
	Sfrp1	Activate	Canonical Wnt signaling	[46]
Fibroblasts	RAD51 BRCA2	Activate	DNA repair	[92]
Epithelial cells Clara cells Tumor cells Endothelial cells Fibroblasts Smooth muscle cells	Cyclin A Cyclin B Cyclin D PLK1 Skp2 Cks1 Cdc25A Cdc25B AURKB Survivin CENPA CENPB	Activate	Cell cycle regulation	[10, 11, 91, 96]
Monocytes/Macrophages	CCR2 CX3CR1	Activate	Recruitment of macrophages/monocytes to induce pulmonary inflammation	[65]
Macrophages	HMMR (CD168)	Activate	Altered migratory cell behavior Pulmonary inflammation	[81, 82]
mDCs	GM-CSFR/CD86 MHCII	Activate	Antigen presentation	[65]
Neutrophils	Eotaxins (CCL11,24) CX3CL1	Activate	Chemoattraction	[65]

### FOXM1 induces replicative immortality via cancer stem cell-like cells

FOXM1 and key proteins of the Wnt/β-catenin pathway were upregulated in CD133 + CD44+ lung cancer stem cells (LCSCs) compared with that in CD133 + CD44- cells [47]. Another finding revealed that FOXM1 upregulation by MnSOD overexpression induced the expression of the cancer stem cell (CSC)-related proteins Oct4, Nanog, and Sox2 in addition to Wnt/β-catenin to maintain self-renewal properties in non-small cell lung cancer stem-like cells (LCSLCs). Inhibiting FOXM1 activation may represent a novel strategy for LCSLCs in the treatment of human NSCLC [48]. In addition, FOXM1 binds to the Sox2 promotor and activates its expression, mediating the renewal of the neural progenitors [49]. Moreover, FOXM1 is involved in the development of SCLC stem cells. MELK inhibitors and T-LAK cell-originated protein kinase (TOPK) inhibitors exert antitumor effects on SCLC by diminishing the FOXM1-mediated transcriptional regulation involved in the proliferation/stemness of CSCs [50, 51].

### FOXM1 enhances invasion and metastasis by promoting epithelial-mesenchymal transition (EMT)

Epithelial-mesenchymal transition (EMT), characterized by decreased E-cadherin expression and increased vimentin expression, mediates cancer cell invasion and metastasis, and transforming growth factor-β1 (TGFβ1) can independently induce EMT in cancer cells [52]. Recent studies have demonstrated that high FOXM1 expression is significantly associated with EMT in NSCLC specimens [29]. FOXM1 promotes the EMT process in lung cancer by directly activating the promoter of the EMT-associated transcriptional factors Snail [53], Twist [47] and Slug [54]. The Wnt/β-catenin pathway upregulates the expression of FOXM1 and that of its direct transcriptional target Twist in CD133 + CD44+ LCSCs, leading to increased migration and invasion ability via CD44-Wnt/β-catenin-FOXM1-Twist signaling [47]. In addition, FOXM1 upregulated by the ERK pathway can mediate TGF-β1-induced EMT in NSCLC. However, knockdown of FOXM1 in NSCLC reversed the phenotype of TGF-β1-induced EMT [52]. Moreover, FOXM1 promotes metastasis by inducing EMT in lung cancer through activation of the AKT/p70S6K pathway, while inhibiting the PI3K/AKT pathway has the opposite effect [29]. Collectively, the ERK-FOXM1-AKT/p70S6K pathway is indispensable for EMT, which is involved in lung cancer cell invasion and metastasis.

Moreover, TGF-β1 treatment significantly decreases miR-134 expression, and the latter directly negatively targets FOXM1, resulting in dramatically increased FOXM1 expression and EMT in NSCLC [55]. MiR-509-5p, miR-149, and miR-361-5p directly bind to the FOXM1 promoter and downregulate its expression, consequently regulating EMT genes, such as E-cadherin, vimentin and MMP2, inhibiting the migratory and invasive capabilities of NSCLC cells [34–36]. Chen et al. provided mechanistic evidence to support the possibility that MnSOD enhances lung adenocarcinoma metastasis predominantly through the FOXM1–MMP2 axis, which mediates cell migration [37]. FOXM1 correlates with poor outcome, and elucidation of its effect on proliferation, invasion, metastasis and replicative immortality provides new insight into understanding the mechanisms of lung cancer progression and drug resistance (Fig. 2).

### FOXM1 is involved in the resistance of lung cancer to anticancer therapy

Clinical treatment of lung cancer consists of surgery, radiotherapy, chemotherapy, immunotherapy, and molecular targeted therapy, and the current therapeutic drugs and their targets are summarized in Additional file 1: Table S1. Because of resistance to treatment, a combination of one or two anticancer drugs is applied, and radiotherapy is often combined with radiosensitizing cytotoxic drugs [56]. Current evidence supports that FOXM1 functions as an attractive chemotherapeutic target involved in DNA damage repair, cell apoptosis signals, and CSCs of various cancers, including lung cancer [57].

Two studies revealed that TKIs, including sorafenib and gefitinib, contributed to aberrant activation of the AKT/FOXM1 pathway (downstream targets include survivin, cyclin B1, SKP2, PLK1, Aurora B kinase, CDC25B, and stathmin1 (STMN1)), coupled with CSC enrichment. Interestingly, blocking the PI3K/AKT pathway and knocking down FOXM1 and STMN1 led to the inhibition of CSC enrichment and consequently enhanced the sensitivity of NSCLC cells to TKIs. Therefore, FOXM1 could be used as a therapeutic target to overcome resistance to TKIs [32, 58].

Vinorelbine (NVB) and Taxol induce mitotic spindle defects, and drug resistance has become a major obstacle to their clinical applications. FOXM1 positively regulates the expression of motor adaptor bicaudal D2 (BICD2); thus, targeting FOXM1 and BICD2 is an effective strategy for sensitizing cells to NVB by enhancing cell proliferation inhibition, mitotic arrest and, subsequently, apoptosis. Interestingly, the combination of DT-13 (saponin monomer 13 of the dwarf lilyturf tuber) and NVB synergistically enhanced cytotoxicity through inhibition of the FOXM1-BICD2 axis in NSCLC cells in vivo and in vitro [59]. Moreover, FOXM1 inhibition enhanced the chemosensitivity of docetaxel-resistant cells to docetaxel by inducing the activation of the c-JNK/mitochondrial signaling pathway to induce apoptosis [60]. The expression of FOXM1 was also significantly higher in cisplatin-resistant cell sublines than in cisplatin-sensitive A549 cells, and inhibition of the expression of FOXM1 reversed the resistance to cisplatin [23]. In addition, the miR-134/FOXM1/multidrug resistance-associated protein 1 (MRP1) signaling pathway provides novel insight into the mechanisms underlying drug resistance [61].

After DNA damage by radiation, FOXM1 expression and phosphorylation are enhanced, thereby mediating the resistance to radiation. Inhibition of FOXM1 decreases the expression of the repair gene XRCC1, p53 phosphorylation and the expression of its downstream target gene p21^Cip1/Waf1^ and increases DNA damage, consequently increasing cell sensitivity to radiation. The study also indicated that FOXM1 functions upstream of p53 in irradiated cells, while MMP-2 inhibition attenuates radiation-induced FOXM1 expression; however, the underlying mechanism needs further investigation [62]. Knockdown of FOXM1 expression decreases the ionizing radiation dose and shortens the clonogenic survival of four NSCLC cell lines (SW1573, A549, H1299, and H322). Strikingly, the inhibition of FOXM1 levels in primary human fibroblasts cannot enhance radiosensitivity [63]. Recently, Xiu et al. reported that FOXM1 promoted radioresistance in lung cancer cells partially by upregulating kinesin family member 20 A (KIF20A) [64]. These results indicated that FOXM1 is upregulated in resistant cancer cells and that reduced expression of FOXM1 enhances their sensitivity to radiation, which may provide a new approach to elevating the sensitivity to treatment in patients with lung cancer. In summary, FOXM1 is overexpressed in cancer cells resistant to TKIs (including sorafenib and gefitinib), NVB, docetaxel, cisplatin and radiation, and inhibiting the expression of FOXM1 is a promising method for lowering resistance to lung cancer treatment.

## The role of FOXM1 in chronic airway disease

Chronic airway disorders, including COPD and asthma, are associated with persistent pulmonary inflammation and goblet cell metaplasia and contribute to significant morbidity and mortality worldwide [65]. The current COPD and asthma therapeutic drugs and their targets are summarized in Additional file 1: Table S1.

### The role of FOXM1 in asthma

Asthma is characterized by persistent pulmonary inflammation, goblet cell metaplasia, variable airflow obstruction, bronchial hyperresponsiveness and lung remodeling [66]. The FOXM1 transcription factor is critical for the proliferation and differentiation of Clara cells during the development of conducting airways [67], and FOXM1 is associated with asthma [65, 68–70]. CCSP-FOXM1 transgenic mice (specific expression of FOXM1-ΔN in Clara cells) exhibited focal airway hyperplasia and increased proliferation of Clara cells during the postnatal period [16], while conditional deletion of FOXM1 from Clara cells (CCSP-FOXM1−/− mice) directly inhibited the transcriptional activity of Sox2 and Scgb1a1, which mediate the differentiation and function of Clara cells, causing squamous and goblet cell metaplasia, dramatically changing airway structure, causing peribronchial fibrosis, and contributing to airway hyperreactivity in adult mice [67].

Evidence that FOXM1 plays an important role in the pathological mechanism of allergen-induced lung inflammation and goblet cell metaplasia in asthma includes the following: (i) Ren X and colleagues demonstrated that pulmonary allergen sensitization induces the expression of FOXM1. (ii) FOXM1 increases the cell surface expression of MHC II and CD86 in myeloid dendritic cells (DCs), leading to increased activation of T cells and increased production of Th2 cytokines (IL-4, IL-5, and IL-13). FOXM1 also recruits eosinophils and macrophages into lung tissue, at least in part by activating eotaxins (CCL11, 24), CCR2, CX3CR1, IL-5, and CX3CL1. Recruitment of the four types of cells mentioned above induces chronic inflammation. (iii) FOXM1 induces the differentiation of Clara cells into goblet cells through direct transcriptional activation of Spdef, a critical regulator of mucus production and goblet cell differentiation. In turn, the upregulation of Spdef inhibits FOXM1 in differentiated goblet cells. (iv) Inhibition of FOXM1 with the ARF peptide or LysM-Cre/Foxm−/− mice (deletion of FOXM1 from myeloid lineage cells, including macrophages, monocytes, DCs, and granulocytes, and from a subset of alveolar type II cells) reduced pulmonary inflammation and airway resistance after house dust mite (HDM) challenge. Therefore, FOXM1 suppression is a promising therapeutic approach in asthma treatment [65]. At the same time, inhalational allergens (such as HDM) can promote ROS generation, while upregulated ROS induced by fatty acid binding protein 4 (FABP4) consequently activates FOXM1, leading to allergic airway inflammation (IL-4, IL-5, and IL-13) and epithelial barrier dysfunction (structural and functional abnormalities of E-cadherin), which are involved in asthma. However, the molecular mechanism involving FABP4 and FOXM1 is unclear [70]. Interestingly, Sun L. et al. identified that inhibition of FOXM1 by RCM-1 reduced IL-13 and STAT6 signaling, prevented the expression of the STAT6 target genes Spdef and Foxa3 in goblet cells, prevented goblet cell metaplasia, decreased airway resistance, and reduced lung inflammation in mice in response to HDM and recombinant IL-13, providing a new potential therapeutic for asthma [69].

Recently, Liu L et al. indicated that sphingosine-1-phosphate (S1P) stimulates airway smooth muscle cell (ASMC) proliferation, migration and contraction by modulating the ROCK/YAP/FOXM1 axis. In addition, silencing FOXM1 reversed the effect of S1P on ASMC functions, suggesting that targeting this pathway might be a potential treatment for asthma [68].

### The role of FOXM1 in chronic obstructive pulmonary disease (COPD)

Increased mucus production by goblet cells is a key feature of chronic respiratory disorders, including COPD. Researchers have also explored whether FOXM1 is overexpressed in the bronchiolar epithelial and inflammatory cells of COPD patients. Interestingly, Spdef is also induced by FOXM1 in goblet cells of the lungs of individuals with COPD [65]. Furthermore, FOXM1 expression is also increased in cigarette smoke (CS)-induced emphysema in mice. These results may enhance our understanding of the CS-induced molecular processes underlying emphysema development in mice and their relevance in human COPD [71]. Further study is needed to reveal the effect of FOXM1 regulators on COPD.

## The role of FOXM1 in the repair of acute lung injury (ALI)

The pathophysiology of both ALI and its potentially devastating form acute respiratory distress syndrome (ARDS) is characterized by increased permeability of the alveolar-capillary barrier, resulting in acute inflammation in the airspace and lung parenchyma, eventually resulting in respiratory function failure [72, 73]. Currently, there is no effective pharmacological treatment for ALI [74].

FOXM1 expression was shown to be markedly increased after BHT injury and to mediate bronchiolar and type II alveolar epithelial cell proliferation throughout the lung repair process [75]. Investigators found that ubiquitous expression of FOXM1 in distinct pulmonary cell types, including alveolar type II epithelial cells, bronchial epithelial and smooth muscle cells, and endothelial cells, accelerates the onset of proliferation in response to BHT lung injury by activating the cell cycle regulatory genes that promote both DNA replication and mitosis [76]. This result indicates that FOXM1 is critical for repair after lung injury.

### FOXM1 promotes restoration of the capillary endothelial barrier

Restoration of the injured vascular endothelial barrier requires both endothelial regeneration and reannealing of adherens junctions (AJs) [77]. Endothelial cell-restricted FOXM1-deficient mice (FOXM1 CKO mice) showed that suppression of FOXM1 inhibited cell cycle progression and cell proliferation and failed to rescue the lung vascular endothelial barrier defects induced by lipopolysaccharide (LPS); these mice also displayed markedly increased mortality [78]. β-catenin is an integral part of the endothelial intercellular AJs [79]; FOXM1 directly binds to the promoter of the human ctnnb1 gene and promotes β-catenin transcription. Furthermore, FOXM1 CKO lung microvessels fail to restore disassembled AJs and ameliorate the leakage caused by PAR-1 activation, and reexpression of β-catenin can rescue defective AJ reannealing. Taken together, these results demonstrate that FOXM1 is indispensable for endothelial proliferation, as it controls cell cycle genes, and the reannealing of endothelial AJs depends on the control of β-catenin transcriptional expression [80]. Another study indicated that the upregulation of FOXM1 and its downstream targets CCNB1, CCNB2 and TOP2A is involved in sepsis-related ARDS. The receptor for hyaluronan-mediated motility (HMMR, also identified as CD168) was first identified as a direct target gene of FOXM1 and may be correlated with the progression of ARDS [81]; Cui et al. identified that CD168 promotes inflammation and fibrosis after ALI [82]. Further investigation of the FOXM1-HMMR axis in the progression of ALI is needed. Meanwhile, transgenic expression of FOXM1 in lung endothelial cells can repair lung vascular injury and reduce morality induced by various sepsis challenges in mice [83]. Huang X et al. showed for the first time that FOXM1 is the critical downstream target of endothelial p110γ and indicated that CXCL12 signaling-activated p110γ (GPCR-dependent p110γPI3K) in endothelial cells mediates FOXM1 upregulation, thereby promoting endothelial regeneration and vascular repair after inflammatory injury induced by endotoxemia and polymicrobial sepsis [84].

Bone marrow-derived progenitor cells (BMPCs) can reanneal endothelial AJs by promoting paracrine S1P release in the inflammatory environment and represent a promising novel approach to addressing ARDS within hours [85]. The endogenous mediators in the pulmonary vasculature include the endothelial FOXM1/β-catenin axis, which mediates endothelial cell proliferation and the assembly of endothelial cell-cell contacts. BMPC therapy failed to repair the lung vascular injury resulting from the endothelial cell-specific inactivation of FOXM1 (FOXM1 CKO) in mice. Exogenous adult stem/progenitor cells elicited effects through endothelial FOXM1 to promote vascular repair, lung inflammation resolution and survival [86]. The anti-inflammatory effect of BMPCs during the injury/acute phase (24 h) is independent of endothelial FOXM1. However, the rapid resolution of lung inflammation induced by BMPCs also requires endothelial expression of FOXM1, which is consistent with previous research showing that FOXM1 is markedly induced in lung endothelial cells but during only the repair phase following sepsis-induced lung injury, while FOXM1 expression is silenced in pulmonary vascular endothelial cells in adult lungs [78, 83].

Collectively, the evidence presented suggests that enhancing FOXM1 levels is an effective means to promote cellular proliferation and repair after lung injury and reveals that activation of the p110γ-FOXM1-β-catenin axis in endothelial cells may represent a novel therapeutic strategy for restoring vascular homeostasis, reducing lung edema and treating inflammatory vascular diseases, such as ALI/ARDS; however, further clinical trials are needed.

### FOXM1 promotes repair of the alveolar barrier

The expression of FOXM1 in type II cells is critical for their proliferation and transition into type I cells and for repairing the alveolar barrier after *Pseudomonas aeruginosa*-induced lung injury [87]. Xia H et al. also suggested that patients with bronchopulmonary dysplasia (BPD) and hyperoxia-exposed mice have increased FOXM1 expression in their lungs, which limits alveolar injury and promotes remodeling. FOXM1 expression in myeloid cell lineages is required to maintain balance between neutrophils and interstitial macrophages, which limits alveolar injury and promotes remodeling in response to prolonged neonatal hyperoxia. LysM-Cre/Foxm−/− mice (deletion of FOXM1 from myeloid cells) display impaired alveologenesis after prolonged hyperoxia. Therefore, pharmacological agents that can modulate FOXM1 function in postnatal lungs could be beneficial for inhibiting hyperoxia-induced inflammation and stimulating lung repair in patients with BPD [88].

## The role of FOXM1 in pulmonary fibrosis

**Fig. 3:**
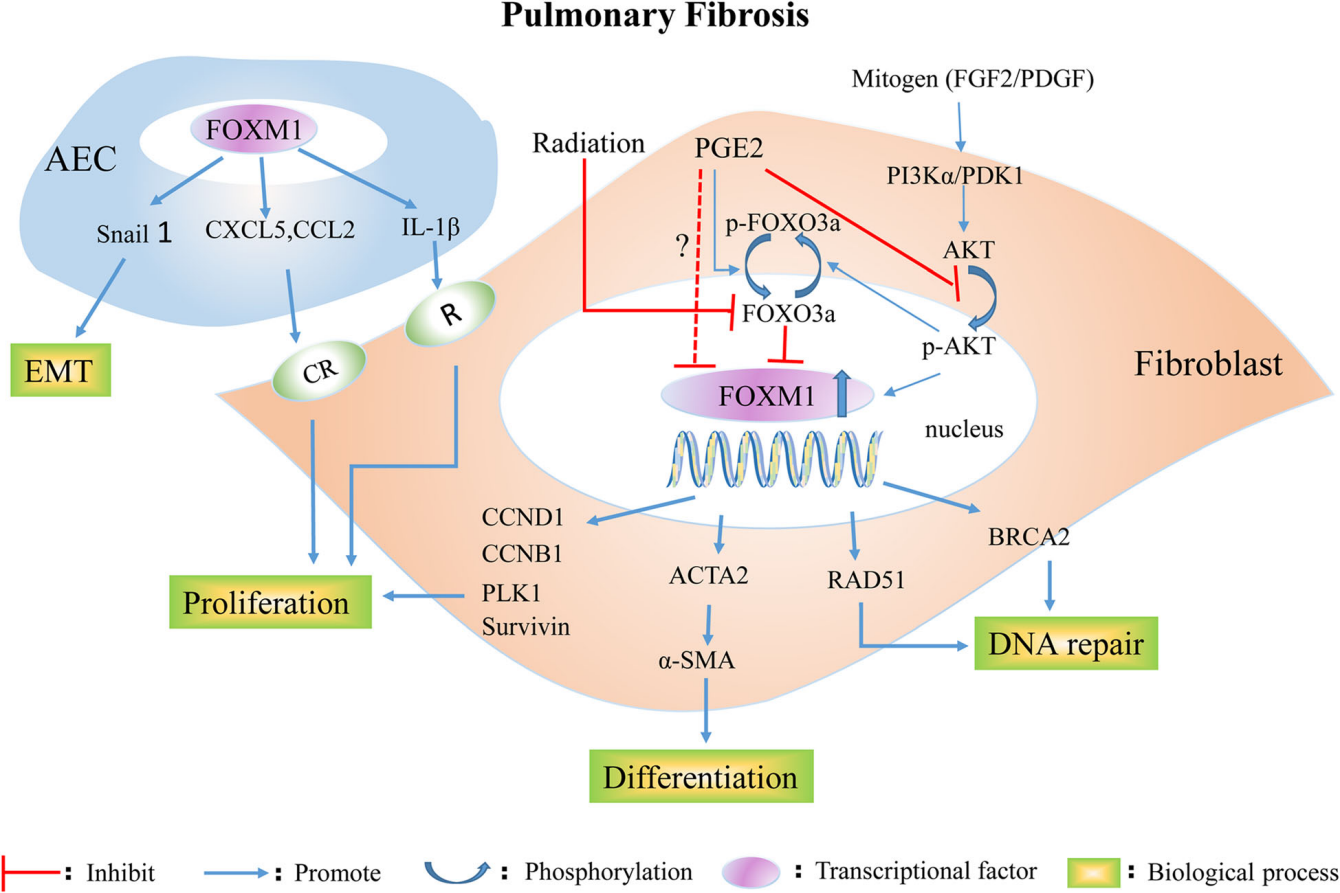
The mechanism regulation of FOXM1 in pulmonary fibrosis. In response to various stimulus, such as inflammatory mediators, radiation and mitogen, FOXM1 induces the epithelial-to-mesenchymal transition in alveolar type II epithelial cells, and activates fibroblasts. Elevated FOXM1 transcription in activated fibroblasts promote the fibroblast proliferation, differentiation and DNA repair, consequently accelerating the process of pulmonary fibrosis. EC: endothelial cell; SMC: smooth muscle cell; CXCL12: chemokine ligand 12; CXCR4: chemokine receptor type 4; ET-1: endothelin-1; IGF-1: insulin-like growth factor-1; MIF: macrophage migration inhibitory factor; NBS1: Nijmegen breakage syndrome 1; PDGF-B: platelet-derived growth factor -B; PGE2: prostaglandin E2; BRCA2: breast cancer-associated gene 2

Pulmonary fibrosis results from dysregulated repair of damaged tissue following a variety of damaging stimuli; chronic inflammation is correlated with cancer therapy (ionizing radiation, chemotherapy, such as bleomycin), pathogen infection, cigarette smoking, PAH, and idiopathic pulmonary fibrosis (IPF) with unknown cause [89]. No specific beneficial drug therapies directed at lung fibrosis have been identified [90]. Activated fibroblasts, which are derived from (i) resident stromal fibroblasts, (ii) bone marrow-derived ‘fibrocytes’ and (iii) alveolar type II epithelial cells (EMT), play a central role in the pathogenesis of pulmonary fibrosis by synthesizing and depositing ECM proteins [9]. FOXM1 promotes the process of pulmonary fibrosis, and the mechanism underlying the regulation of FOXM1 in pulmonary fibrosis is depicted in Fig. 3.

The expression of FOXM1 is elevated in lung fibroblasts isolated from IPF patients and the bleomycin-treated standard mouse model [91]. Researchers have indicated that mitogens (FGF2 and PDGF) increase FOXM1 expression via a PI3Kα/PDK1/AKT activation pathway, and the upregulated expression of FOXM1 promotes lung fibroblast proliferation by inducing the expression of proliferation-associated genes, such as CCND1, CCNB1, PLK1, and BIRC5 (also known as survivin). Interestingly, prostaglandin E2 (PGE2) was identified as the endogenous inhibitor of FOXM1 for the first time (via an EP2/cAMP pathway involved in AKT and FOXO3a phosphorylation). Specific deletion of FOXM1 from activated fibroblasts in FOXM1fl/fl Col1a2-Cre-ER(T)+/0 mice protects mice from bleomycin-induced fibrosis. In addition, inhibition of FOXM1 during the fibrotic phase was able to attenuate bleomycin-induced pulmonary fibrosis independent of widespread alveolar cell apoptosis. This finding shows that FOXM1 is critical for both myofibroblast differentiation (ACTA2, encoding α-SMA) and apoptosis resistance in response to TGF-β stimulation (Fig. 3) [91]. The activation of FOXM1 occurs secondary to a reduction in FOXO3a expression, and subsequent upregulation of the DNA repair proteins RAD51 and breast cancer-associated gene 2 (BRCA2) protects human IPF primary fibroblasts from radiation-induced death. This observation demonstrated that decreased radiosensitivity in IPF fibroblasts occurs through a FOXO3a-dependent FOXM1/RAD51–BRCA2 pathway [92].

Based on gain/loss-of-function mouse models, Balli D et al. confirmed that transgenic activation of FOXM1 in alveolar epithelial cells (SP-C–rtTA^tg^/^−^ / tetOFOXM1-ΔN^tg^/^−^ or epiFOXM1-ΔN mice) accelerates fibrosis after irradiation by (i) directly activating the snail1 promoter, consequently inducing EMT, and (ii) increasing the expression of CCL2, CXCL5, IL-1β, and, consequently, inflammatory mediators, inducing fibroblast proliferation (Fig. 3). In contrast, conditional deletion of FOXM1 from the respiratory epithelium (Spc-rtTA/tetO-cre/FOXM1fl/fl, termed epiFOXM1 KO mice) protected mice from radiation-induced fibrosis [9]. Altogether, these results suggest that FOXM1 may be a novel therapeutic target for the treatment of fibrotic diseases of the lung and, potentially, of other organs.

## Role of FOXM1 in pulmonary arterial hypertension (PAH)

PAH is a devastating disease that is characterized by the phenotypic change in pulmonary artery smooth muscle cells (PASMCs) from a contractile or differentiated phenotype to a proliferative or dedifferentiated phenotype, PASMC proliferation and vascular remodeling. The drugs that are currently used for the treatment of PAH regulate the disrupted nitric oxide-sGC-cGMP signaling pathway (Additional file 1: Table S1). Hypoxia is a well-known stimulus for the development of PAH, and both hypoxia-inducible factor (HIF)-1a and HIF-2a are implicated in the pathogenesis of PAH [93–96]. The FOXM1 promoter contains HIF response elements, and hypoxia-induced PASMC proliferation is controlled by FOXM1 [94]. FOXM1 is overexpressed in PASMCs from PAH patients and animal models [97]. Interestingly, several prohypertensive factors, including hypoxia, have been shown to reduce miR-204 expression in PASMCs [97]; downregulation of miR-204 in PAH PASMCs contributes to enhanced FOXM1 expression [98].

HIF-2a is required for the hypoxia-stimulated expression of FOXM1 and the induced proliferation of human pulmonary artery smooth muscle cells (HPASMCs), whereas HIF-1a participates in FOXM1 regulation under normal conditions in HPASMCs. However, overexpression of FOXM1 is not sufficient to induce HPASMC proliferation during normoxia [94]. HIF-1 directly binds to the FOXM1 promoter and upregulates the expression of FOXM1, and downstream target genes mediate cancer cell proliferation under hypoxic conditions [99]. This difference can be mainly explained by the fact that the FOXM1 promoter contains HIF response elements. Both HIF isoforms regulate FOXM1, share some common target genes and functions, and vary in their tissue distribution and response to different degrees of O2 concentration [94].

**Fig. 4:**
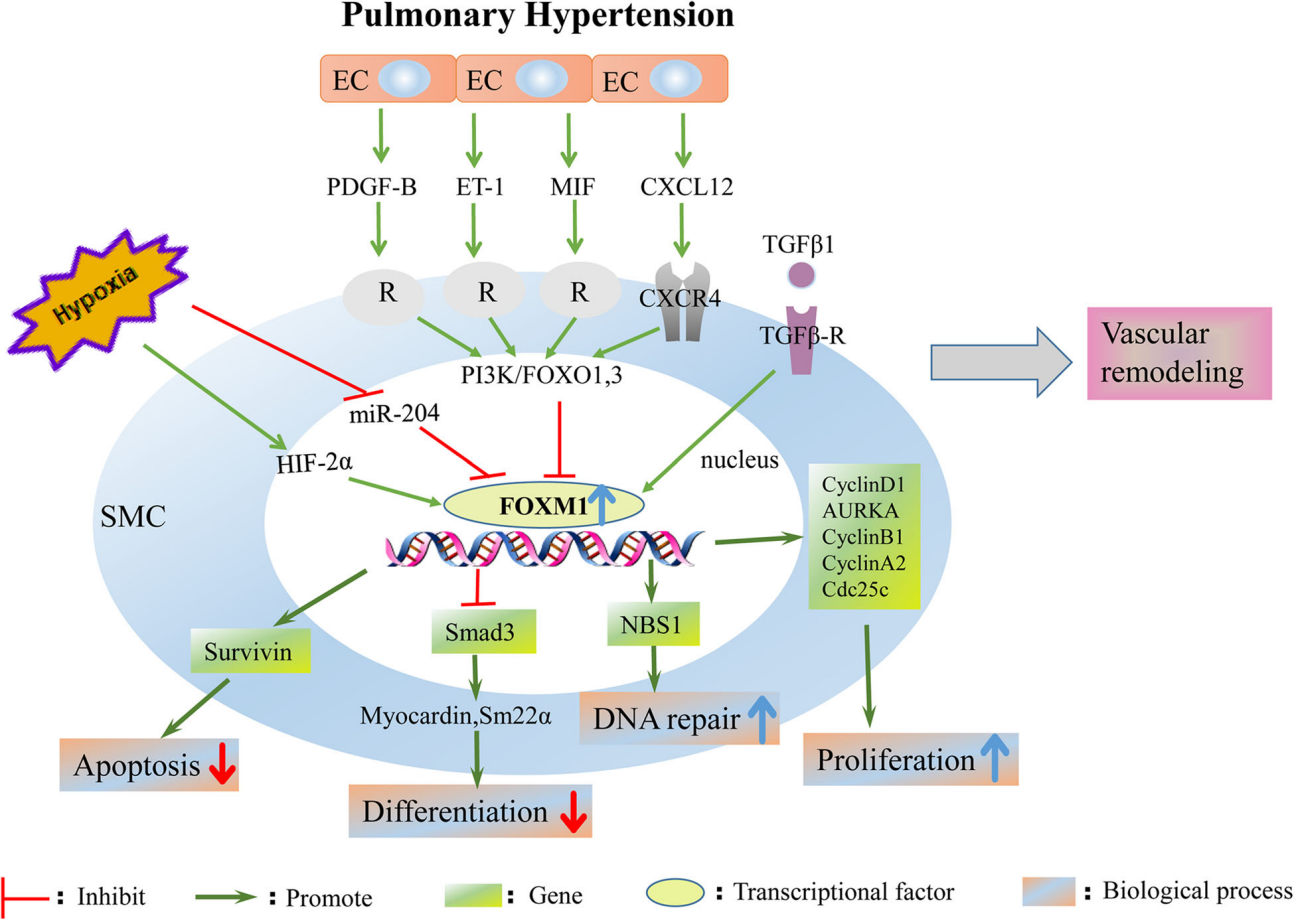
FOXM1 promotes the initiation and progression of pulmonary arterial hypertension. Hypoxia, elevated growth factor and inflammatory cytokine from endothelial cells induces upregulated expression of FOXM1 in smooth muscle cells, and the increased FOXM1 targets on numerous pathways, such as inhibiting SMC apoptosis and differentiation, increasing SMC DNA repair and proliferation, consequently promoting vascular remodeling of PAH

Multiple factors (PDGF-B, CXCL12, ET-1 or MIF) derived from dysfunctional endothelial cells induce FOXM1 expression in SMCs and activate FOXM1-dependent SMC proliferation, which contributes to vascular remodeling and PAH [100]. In addition, downregulated FOXO1 and FOXO3 in PAH PASMCs may contribute to vascular remodeling by decreasing the inhibition of FOXM1 transcription [101]. PI3K/FOXO signaling is likely the common mechanism mediating the enhanced expression of FOXM1 induced by paracrine factors from endothelial cells (Fig. 4) [100]; future studies are warranted to investigate this possibility. FOXM1-mediated PASMC proliferation and dedifferentiation are also correlated with decreased TGF-β/Smad3-dependent signaling; however, further study is needed to uncover the molecular mechanisms underlying this observation [96]. Moreover, FOXM1 enhances DNA repair capacity by stimulating the expression of NBS1 and reduces apoptosis by enhancing the expression of survivin, contributing to SMC hyperproliferation and disease progression [98].

Overexpression of FOXM1 enhanced hypoxia-induced pulmonary artery remodeling and right ventricular hypertrophy [96]. In contrast, inhibition of FOXM1 by thiostrepton reduces PAH PASMC proliferation, restrains severe PAH and reduces right ventricle fibrosis in the monocrotaline- and Sugen/hypoxia-challenged rat models [97, 100]. Constitutive knockdown (sm-FOXM1+/−) or inducible knockout of FOXM1 in SMCs (sm-FOXM1−/− mice) prevented or reversed hypoxia-induced lung vascular remodeling in PAH in vivo, respectively [96]. In addition, deletion of Cxcr4, a well-known receptor of CXCL12, inhibited FOXM1 expression in SMCs, protecting mice against hypoxia-induced PAH [100].

The FOXM1 regulatory network in PAH is described in Fig. 4. The vascular wall in various environments with elevated growth factor and inflammatory cytokine levels was explored in PAH [102]. Because the current study was designed to explore the role of FOXM1 in SMCs and the interaction with endothelial cells, it is possible that other cell types (including adventitial fibroblasts and inflammatory cells) as well as the interactions between these cells and SMCs are also involved in the pathological remodeling of the distal pulmonary artery, and this possibility needs to be further addressed [98, 103]. Therapies targeting FOXM1 may provide novel insights into the treatment of PAH.

## Regulators of FOXM1 and potential implications of targeting FOXM1

The above mentioned studies indicate that FOXM1 is critical in the development of pulmonary disease and that FOXM1 is potentially a promising therapeutic target. The FOXM1 regulators can be divided into inhibitors and activators. As humans are an integral body, a mechanism for precisely regulating FOXM1 expression is urgently needed.

Inhibiting the expression of FOXM1 in hyperproliferative disease is a potential treatment method, as evidenced above. There are 8 types of FOXM1 inhibitors, including the thiazole antibiotics siomycin A and thiostrepton, thiazolidinediones, diarylheptanoids, peptide 9R-P201, FDI-6, RCM-1, honokiol, and FOXM1 Apt [4]. In addition, PGE2 [91] and HSP70 [104, 105] were identified as endogenous inhibitory substances of FOXM1. Animal models have shown that FOXM1 inhibitors or the genetic deletion of FOXM1 are beneficial during intervention. However, various challenges, which have been well reviewed in previous work [4] and include targeted drug delivery, overcoming immune responses, evaluating drug toxicity, passing comprehensive preclinical studies on drug efficacies, clarifying the interactions among the four isoforms and determining whether isoform switches exist, must be addressed before these interventions can enter into clinical trials.

Special attention must be paid before FOXM1 can be translated into a specific therapeutic target for lung cancer patients. First, the heterogeneity of lung cancer makes using several targeted drugs in combination reasonable [106], especially in combination with low-dose chemotherapy in resistant models [63]. And developing methods to efficiently explore better combinations of the current drugs targeting lung cancer is necessary [106]. However, targeting FOXM1 is an effective therapeutic strategy for the treatment of lung cancer and COPD in mice. Second, the majority of lung cancer patients, long-term smokers, typically harbor significant smoking-induced comorbidities, such as COPD, coronary heart disease and vascular problems [107]. The efficacy of FOXM1 intervention in lung cancer patients who also have COPD or other problems described above is unclear, and further studies are needed. Importantly, the expression of FOXM1 in premalignant lesions and indeterminate pulmonary nodules may help it serve as a therapeutic target in clinical trials [108]. On the other hand, based on the specificity of the lung structure, the route of local administration, such as intrathoracic injection and inhalation therapy, should be taken into consideration.

Some findings indicate that downregulation of FOXM1 expression fails to restore the damage caused by lung injury [78, 80] and that activating FOXM1 expression is an effective therapeutic strategy because of ineffective DNA repair [84, 86, 88]. Therefore, pharmacological agents that can activate FOXM1 function should not be neglected, and these agents represent a future research direction for our group.

On the other hand, additional strategies can be explored to identify novel and specific mechanisms for regulating FOXM1, such as (i) altering FOXM1 nuclear localization, nuclear export or protein–protein/RNA interactions with activating kinases and coactivator proteins and (ii) mimicking FOXM1-repressive proteins [105, 109] and validating their use in clinical practice. However, the biological effects of targeting transcription factors are diverse, and further studies are warranted to fully dissect the different mechanisms involved in FOXM1 regulation.

## Conclusion and future perspectives

In summary, FOXM1 is a critical transcriptional regulator of alveolar epithelial cells, airway epithelium, endothelial cells, smooth muscle cells, and inflammatory cells (mDCs, monocytes, macrophages, and neutrophils, except lymphocytes) during embryogenesis and the development of pulmonary disease, as summarized in Table 1. Targeting the expression of FOXM1 is a promising potential therapeutic method for lung disease, and all transgenic mouse models are described in Table 2.

**Table 2:** FOXM1 transgenic mouse models used in the study of pulmonary diseases

Mouse models	Expression of FOXM1	Cells	Consequences of the models	Reference
Mx-Cre FOXM1^−/−^ mice	Deletion	All cell types	60% reduction in medium-sized (0.5–1 mm) lung adenomas; no large lung adenomas > 1 mm in size;84% of lung tumors exhibited strong FOXM1 nuclear positivity; 16% FOXM1-negative tumors were significantly smaller in size	[25]
Rosa26-FOXM1 transgenic mice	Overexpression	All cell types	Persistent pulmonary inflammation increased the total number and diameter of lung adenomas	[44]
SPC–rtTA^tg/−^/ TetO-Cre^tg/−^/FOXM1^fl/fl^ mice termed epFOXM1^−/−^ mice, epiFOXM1 KO mice	Conditional knockout	Specifically in lung epithelial cells	Reduced the number (5-fold) and size of lung tumors prior to or even after tumor initiation	[9, 45]
SP-C–rtTA^tg/−^ / tetOFOXM1-ΔN^tg/−^ mice termed epiFOXM1-ΔN mice	Activated FOXM1-ΔN mutant	Epithelial cells	Enhanced radiation-induced pulmonary fibrosis	[9]
SPC-rtTA/TetO-Kras^G12D^	Mutant Kras^G12D^transcript	Respiratory epithelial cells	Activated Kras alone is sufficient to induce formation of lung adenocarcinomas	[24]
SPC-rtTA/TetO-GFP-FOXM1-ΔN/TetO-Kras mice, termed epFOXM1/ep Kras	Activated FOXM1-ΔN mutant and Kras	Respiratory epithelial cells	Tumor sizes are larger than those in epKras mice; FOXM1-ΔN cooperates with activated Kras to accelerate lung tumor growth	[16]
epKras^G12D^/epFOXM1^−/−^ mice	Mutant Kras^G12D^ transcript but deletion of FOXM1	Lung epithelial cells	Prevented the initiation of lung tumors; reduced the number and size of lung tumors; single lung tumors were positive for FOXM1	[24]
Tie2-Cre/FOXM1^fl/fl^ mice termed enFOXM1^−/−^ mice	Deletion	Endothelial cells	Increased lung inflammation and activation of canonical Wnt signaling; increased the lung tumor number and size	[46]
CCSP-rtTA/ TetO-GFP-FOXM1-ΔN mice, termed CCSP-FOXM1 mice	Activated FOXM1-ΔN mutant	Clara cells	Induced airway hyperplasia at sites expressing the transgene	[16]
CCSP-FOXM1^−/−^ mice	Conditional deletion	Clara cells	Reduced pulmonary inflammation and decreased airway resistance after HDM challenge	[67]
FOXM1^fl/fl^ Col1a2-Cre-ER (T)^+/0^ mice	Selective deletion	Activated fibroblasts	Reduced alveolar infiltration and collagen deposition; attenuated bleomycin-induced pulmonary fibrosis even during the fibrotic phase	[91]
sm-FOXM1+/− mice	Constitutive knockdown	Smooth muscle cells	Inhibited hypoxia-induced PH and reversed existing vessel remodeling in hypoxic mice	[96]
sm-FOXM1^−/−^ mice	Knockout	Smooth muscle cells	Induced embryonic lethality in mice	[96]
LysM-Cre/FOXM^−/−^mice	Deletion	Myeloid-derived inflammatory cells	Reduced pulmonary inflammation and airway resistance after HDM challenge	[65]

Our review is the first to summarize the multifaceted roles of FOXM1 in pulmonary diseases. Previous studies have mainly focused on the relationships between FOXM1 and various cancers. However, because of the complex interaction network of FOXM1 with a large number of genes, the beneficial effects of FOXM1 inhibition observed both in vitro and in vivo are likely the result of a cumulative effect on numerous target pathways. Although much work has been done, in addition to the development of new targeted therapeutics, several technological advances are needed to allow the estimation of disease risk and to ascertain which patients should be treated. These advances are essential for expediting the initiation of clinical trials for promising therapeutics.

## Additional file

Additional file 1: **Table S1.** Drugs for the clinical treatment of pulmonary diseases [110–119]. (DOCX 36 kb)

## Abbreviations

AJs: Adherens junctions
ALI: Acute lung injury
ARDS: Acute respiratory distress syndrome
ASMC: Airway smooth muscle cell
AURKB: Aurora B kinase
BICD2: Bicaudal D2
BMPCs: Bone marrow-derived progenitor cells
BPD: Bronchopulmonary dysplasia
BRCA2: Breast cancer-associated gene 2
CENPA: Centromere protein A
CENPB: Centromere protein B
COPD: Chronic obstructive pulmonary disease
Cox-2: Cyclooxygenase-2
CS: Cigarette smoke
CSC: Cancer stem cell
CXCL12: Chemokine ligand 12
DCs: Dendritic cells
EC: Endothelial cell
EMT: Epithelial-mesenchymal transition
ET-1: Endothelin-1
FABP4: Fatty acid binding protein 4
Flk-1: Vascular endothelial growth factor receptor 2(VEGFR2)
FOXM1: Forkhead box M1
HDM: House dust mite
HIF: Hypoxia-inducible factor
HMMR: Receptor for hyaluronan-mediated motility
HPASMCs: Human pulmonary artery smooth muscle cells
IGF-1: Insulin-like growth factor-1
IPF: Idiopathic pulmonary fibrosis
JNK: Jun N-terminal kinase
KIF20A: Kinesin family member 20 A
LCNECs: Large cell neuroendocrine carcinomas
LCSCs: Lung cancer stem cells
LCSLCs: Lung cancer stem-like cells
LPS: Lipopolysaccharide
MCA/BHT: 3-methylcholanthrene/butylated hydroxytoluene
mDCs: Myeloid dendritic cells
MIF: Macrophage migration inhibitory factor
MMP-12: Matrix metalloprotease-12
MnSOD: Manganese superoxide dismutase
NBS1: Nijmegen breakage syndrome 1
NF-KB: Nuclear factor-KB
NSCLC: Non-small cell lung cancer
NVB: Vinorelbine
PAH: Pulmonary arterial hypertension
PASMCs: Pulmonary artery smooth muscle cells
PDGF-B: Platelet-derived growth factor-B
PGE2: Prostaglandin E2
PH: Pulmonary hypertension
PLK1: Polo-like kinase1
ROS: Reactive oxygen species
S1P: Sphingosine-1-phosphate
SCLC: Small cell lung cancer
Sfrp1: Secreted frizzled-related protein 1
SHH: Sonic hedgehog
SM: Sulfur mustard
SMCs: Smooth muscle cells
STAT6: Signal transducer and activator of transcription 6
STMN1: Stathmin1
TGFβ1: Transforming growth factor-β1
TKIs: Tyrosine kinase inhibitors
