# Supplementary material for: The multifaceted roles of FOXM1 in pulmonary disease
Source: Cell Commun Signal. 2019 Apr 16;17:35. doi: 10.1186/s12964-019-0347-1 (PMC6469073; doi:10.1186/s12964-019-0347-1)
Supplement: Supplementary file 1 — Table S1. Drugs for the clinical treatment of pulmonary diseases [110–119]. (DOCX 36 kb) [file 12964_2019_347_MOESM1_ESM.docx]

Additional file 1: **Table S1** Drugs for the clinical treatment of pulmonary diseases

| Pulmonary disease | Drugs | Mechanism | Reference |
| --- | --- | --- | --- |
| Lung cancer | Erlotinib gefitinib, afatinib osimertinib | Target EGFR | (111) |
|  | Crizotinib | Target ALK, ROS1 and MET | (111, 112) |
|  | Ceritinib alectinib brigatinib | Target ALK | (111) |
|  | Platinum compounds etoposide | Damage DNA | (113) |
|  | Docetaxel paclitaxel vincristine | Antimicrotubule | (113) |
|  | Pemetrexed | Inhibit thymidylate synthase | (113) |
|  | Gemcitabine | Inhibit ribonucleotide reductase Incorporate into DNA | (113) |
|  | Fluoropyrimidines | Antimetabolite | (113) |
| COPD | Salbutamol salmeterol formoterol | Activate β2 receptor | (114, 115) |
|  | Ipratropium bromide | Antagonize muscarinic | (114, 115) |
|  | Inhaled corticosteroids | Inhibit inflammatory cells of airway mucosa | (114, 115) |
| Asthma | Inhaled corticosteroid | Inhibit inflammatory cells of airway mucosa | (116) |
|  | Salbutamol salmeterol formoterol | Activate β2 receptor | (116) |
|  | Montelukast zafirlukast | Antagonize leukotriene receptor | (117) |
|  | Omalizumab | IgE-blocking monoclonal antibody | (117) |
|  | Mepolizumab reslizumab | Interleukin-5 monoclonal antibodies | (118) |
| ALI | No effective pharmacological treatment | | (75) |
| Pulmonary fibrosis | No effective pharmacological treatment | | (91) |
| PAH | Sildenafil tadalafil | Inhibit phosphodiesterase type 5;  Prevent the degradation of cGMP | (119) |
|  | Adempas | Stimulate soluble guanylate cyclase; Increase cGMP production | (119) |
|  | Bosentan, ambrisentan | Antagonize endothelin receptor | (119, 120) |
